# Targeted enamel remineralization with mineral-loaded starch particles

**DOI:** 10.1016/j.jfscie.2024.100041

**Published:** 2024-11-20

**Authors:** Nathan A. Jones, Li-Chi Pan, Susan E. Flannagan, Kai A. Jones, Lyudmila Lukashova, Lucas Wightman, Sywe-Ren Chang, Glenn Jones, Livia M. A. Tenuta, Carlos González-Cabezas, Brian H. Clarkson, Wendy Bloembergen, Steven Bloembergen

**Affiliations:** a GreenMark Biomedical Inc, East Lansing and Ann Arbor, MI;; b School of Dentistry, University of Michigan, Ann Arbor, MI;; c Department of Radiology, Montefiore Medical Center, Bronx, NY;; d Center for Craniofacial Regeneration, School of Dentistry, University of Pittsburgh, Pittsburgh, PA;; e School of Medicine, Saba University, Devens, MA.

**Keywords:** Caries, nanotechnology, remineralization, noninvasive treatment, noncavitated carious lesions, esthetics, targeted delivery

## Abstract

**Background.:**

Noninvasive caries treatments work topically, which may limit efficacy. The authors hypothesized that an alternative approach using mineral-loaded particles designed to target the subsurface of noncavitated caries lesions could be advantageous. This study shows in vitro proof-of-concept.

**Methods.:**

Mineral-loaded cationic starch (MLCS) particles were prepared, containing calcium, phosphate, and fluoride to provide fluoride-plus (FP) and fluoride-free (FF) alternatives. Particles were characterized for mineral loading and release. MLCS-FP and -FF treatments vs 1,000 ppm fluoride and deionized water controls were evaluated on natural smooth-surface caries lesions (n = 15 per group) after a 20-day protocol with immersion in artificial saliva with amylase and acid challenge. Treatment efficacy was assessed by microcomputed tomography, labeled fluorescence imaging, and blinded qualitative visual assessment.

**Results.:**

In aqueous suspension and absent amylase, particles showed sustained mineral ion release. The tomographic evaluation found significant (multivariable regression analysis, *P* < .05) restoration of lesion mineral density by MLCS-FP and MLCS-FF (42.9% and 38.6%, respectively) vs fluoride and negative controls (7.4% and −18%, respectively), particularly for the lesion subsurface (13.8% [13.0%], 15.9% [9.4%], −2.2% [7.3%], and −1.8% [4.0%] relative hydroxyapatite density for 0.25 through 0.45 μm lesion depth for FP, FF, fluoride, and deionized water, respectively). Visually reduced white opacity (Fisher exact test, P = .038, MLCS-FF vs fluoride) and labeled fluorescence (analysis of variance, *P* < .05 for MLCS-FF [75.4%], MLCS-FP [75.7%], fluoride [64.1%] vs negative control [−0.2%]) were observed.

**Conclusions.:**

These foundational studies show the potential of mineral-loaded starch particles to remineralize enamel as a new approach to treating early caries by subsurface targeted mineral delivery. The in vitro study results indicated that targeted particles improved treatment efficacy, with the data supporting the superiority of MLCS-FP and FF formulations over control conditions for subsurface remineralization and visual esthetic.

## Graphical Abstract

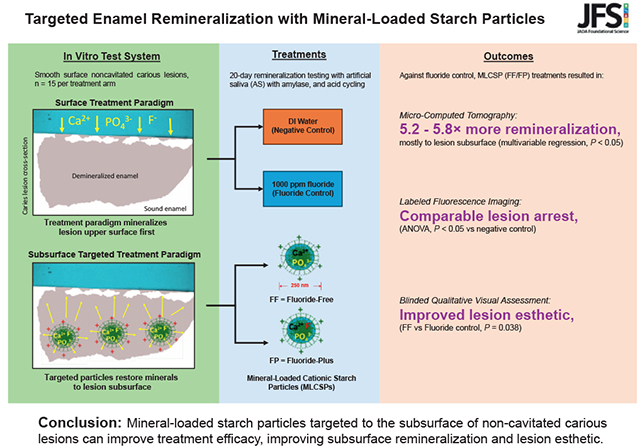

## Introduction

Caries is the most prevalent chronic disease worldwide, and over their lifetimes, nearly everyone will develop caries lesions.^1–4^ Approximately 42% of children and 25% of adults have untreated caries,^1,3^ with potential complications of pain, infection, tooth loss, poor quality of life, and, in rare cases, death.^5–10^ Surgical treatment of caries initiates an irreversible restorative cycle of restorations, then crowns, and as patients age, eventually leading to tooth loss and replacement with dentures or dental implants.^11–13^

The caries process is dynamic; early-stage noncavitated caries lesions, also known as incipient or white-spot lesions, can be reversed with better hygiene and applications of remineralization agents such as topical high-fluoride treatments or fluoride toothpastes.^11^ This has effected a paradigm shift to emphasize prevention and earlier intervention.^14–18^ Disease management is moving from surgical treatments to a medical approach, aiming for better oral health outcomes.^19,20^ Consequently, some early white-spot lesions become inactive or arrested and do not require further treatment,^14,21,22^ because surface porosity has been blocked or reduced by mineral or protein deposition. However, caries progression rates remain higher for inactive noncavitated surfaces than sound surfaces,^23^ suggesting that caries arrest without subsurface remineralization makes these surfaces more vulnerable to new or continued caries processes.

**Figure 1:**
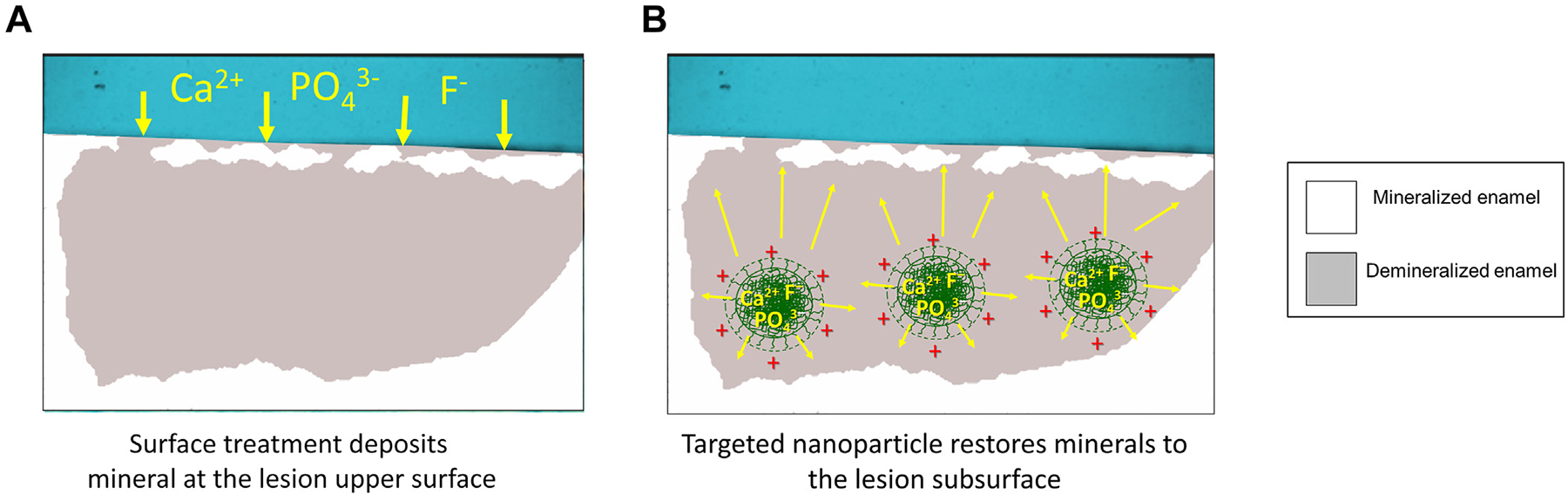
**A**. The concept of a surface treatment paradigm is illustrated with a diagram of the cross-section of a noncavitated caries lesion, with higher mineral treatment concentrations limited to the lesion surface. **B**. The subsurface targeted treatment paradigm concept is illustrated with guided mineral delivery using targeted particles, which shift the axis of treatment. Ca^2+^: Calcium cation. F^−^: Fluoride ion. PO_4_^3^^−^: Phosphate ion.

As shown in a concept figure (Figure 1), although demineralized, the typical noncavitated caries lesion has a more highly mineralized surface overlying more substantial subsurface demineralization.^24^ As a result of this architecture, treatments deliver minerals and fluoride at high concentrations to the surface layer and depend on diffusion through this layer to remineralize the dominant subsurface lesion (Figure 1A). As mineral deposition seals this surface layer, diffusion is impeded, leaving the dominant subsurface lesion untreated. This treatment approach may perpetuate caries progression because, without improved oral hygiene, the lesion surface may reopen, and bacterial acids again reach the subsurface, resuming disease progression and ultimately producing cavitation, requiring surgical restoration.^25^ In contrast, a subsurface targeted delivery approach suggests an alternative treatment paradigm with the release of therapeutic mineral ions and fluoride at high concentrations in the lesion subsurface (hypothesis in Figure 1B). Consequently, remineralization would occur primarily in the subsurface, which could theoretically restore more minerals to the lesion.

In this context, nanotechnology has emerged as a field with potential uses. Nanoscale and submicroscale particles exhibit unique physicochemical properties because of the high surface area to volume ratio, tunable surface characteristics, and capacity for functionalization.^26–28^ These properties make such particles attractive candidates for targeted delivery systems in the management of caries, allowing for site-specific drug release and enhanced therapeutic efficacy.

Research developed and validated the targeting capabilities of fluorescently labeled starch nanoparticles designed to target the subsurface of active caries lesions.^29^ These particles infiltrate the lesion through open surface porosities and adhere to the subsurface to illuminate pathways in which acids from cariogenic biofilms demineralize the enamel, predominantly between hydroxyapatite crystal rods. The particles agglomerate within the entire interior of the lesion, thereby defining the intricate, interconnected subsurface morphology of the caries lesion, as shown with 2-photon micrographs.^29^ The particles’fluorescence is illuminated with a dental curing lamp, and the starch-based chemistry allows for the degradation of these particles with amylase in saliva. This nanoparticle-based targeting of caries was first shown in vitro, with high sensitivity, specificity, and reproducibility compared with histologic reference standard^30^ and was further verified clinically with a study confirming high sensitivity and specificity compared with clinical assessment using the International Caries Detection and Assessment System classification (ICDAS).^16,31^ Additional research applied image analysis and machine learning to fluorescent images as a diagnostic tool and to facilitate monitoring of caries progression and remineralization.^30,32^

We propose an extended hypothesis for typical noncavitated caries lesions, suggesting that as topically applied calcium, phosphate, and fluoride ions diffuse into active initial caries lesions, they are likely to become entrapped within the intricate network of small pores of the lesion surface layer because of their strong polarity and high affinity for precipitation. In contrast, less polar materials like hydroxyapatite (HA), fluorohydroxyapatite (FA), amorphous calcium phosphate (ACP), or specifically targeted particles could potentially diffuse across the surface layer and navigate the entire subsurface of caries lesions, influenced by the structural porosity of the lesion as well as the physical dimensions of the materials. However, HA, FA, and ACP carry negative surface charges,^33^ which might hinder their ability to penetrate and persist within negatively charged caries lesions, as observed with anionic starch nanoparticles that did not, compared with cationic particles that did.^29^

Consequently, we devised a strategy involving sustained-release mineral ion components that are encapsulated within targeted cationic starch particles based on prior reported chemistries.^29^ These particles possess an inherent electrostatic driving force, enabling them to penetrate caries lesions and to persist within subsurface pores, thereby delivering ionic minerals over time, nucleating HA or FA crystal formation in situ. Our study aimed to show a proof-of-concept for targeted mineral-loaded cationic starch (MLCS) particles designed for sustained mineral delivery as a treatment for noncavitated caries lesions. We prepared and characterized submicrometer MLCS particles and performed remineralization testing on natural caries smooth-surface lesions in extracted human teeth.

## Methods

### Materials

MLCS particles (provided by GreenMark Biomedical Inc) were prepared from food-grade starch in either fluoride-plus (FP) or fluoride-free (FF) compositions. These particles are approximately 150 through 350 nm and contain cationic starch with chemical modifications to incorporate phosphate and calcium, with or without fluoride. MLCS-FP and MLCS-FF particles are lyophilized powders that are to be suspended before use. For fluorescence imaging experiments, LumiCare Caries Detection Rinse (LC Rinse) (Greenmark Biomedical Inc), a commercial rinse based on fluorescent cationic starch-based particles, was used.

### Mineral loading and release study

To estimate total loading, particles were extracted into 0.5 M hydrochloric acid (HCl), twice, for 1 hour each time. The first extraction used 10 mg MLCS particles/mL of 0.5 M HCl; the second extraction used twice the volume of HCl (ie, 5 mg MLCS particles/mL of 0.5 M HCl).

To determine mineral release, particles were dispersed in 5 mL of deionized (DI) water at a concentration of 1 mg/mL within a dialysis membrane (Float-A-Lyzer G2, 10 kDa, Spectrum), which was then immersed in 45 mL of DI water at 37 °C under agitation at 60 rpm (n = 2). Aliquots (0.5 mL) of the dialysate were removed at each measurement time point for up to 2 weeks. Both loading and release experiments were repeated in duplicate, with triplicate measures for each collected sample. Mineral measurement assays were Arsenazo III colorimetric method for calcium detection, malachite green colorimetric method for inorganic phosphorus detection, and ion-specific electrode using TISAB II for fluoride detection.^34,35^ Calibration curves for all measurements were prepared using standards under the same sample conditions (eg, 0.5 M HCl for the acidic samples). Release profiles were developed using calculated mineral concentrations, which were plotted against time.

### X-ray diffraction study

Prepared MLCS-FF and MLCS-FP particles were analyzed by x-ray diffraction (XRD) using a Rigaku Ultima 4 diffractometer with a beam voltage of 40 kV and a current of 44 mA using a copper k-alpha emission source. The experimental setup used a glass sample tray, Bragg-Brentano geometry, with a scan step size of 0.05° per step and a scan speed of 0.5° per minute. The background signal was subtracted, and peak-fitting software analyzed the residual collected scans.

### Remineralization studies

To better simulate in vivo conditions, testing was performed on smooth-surface noncavitated natural caries lesions. Deidentified human teeth, extracted for other clinical reasons from a pool of anonymous donors, were obtained from the University of Michigan School of Dentistry in compliance with the institutional review board.

#### Natural caries lesions model

Smooth-surface natural noncavitated caries lesions (ICDAS severity score of 2) were selected by an ICDAS-trained cariologist (S.E.F.) from extracted human teeth (15 per group for a total of 60). Teeth with dental fluorosis, tetracycline stain, hypoplasia, and dental sealants were excluded. Baseline micrographs were obtained, and the teeth were initially evaluated by fluorescence imaging after a 30-second immersion in LC Rinse to determine lesion activity. Fluorescence indicates accessible subsurface porosity with a high correlation to ICDAS activity assessment.^31^ Image analysis, enhanced with an artificial intelligence (AI) fluorescent pixel extraction model,^36^ was performed to determine the number and intensity of fluorescent pixels. After a 24-hour rinse-out in artificial saliva with amylase to degrade and wash away the fluorescent starch particles and subsequent application of an acid-resistant varnish to coat half of the visible lesion, the teeth underwent a 20-day remineralization protocol. Tooth samples were numbered and assigned randomly into treatment arms.

Gel formulations of 1.0% w/w aqueous dispersions of MLCS-FP and MLCS-FF compositions were combined with noninteracting excipients, which included a thickener and stabilizers, and then compared with a 1,000 ppm of fluoride (from sodium fluoride in DI water) positive control and a DI water negative control. Prepared tooth samples underwent a 20-day accelerated remineralization protocol, which included 4 daily 4-minute treatments, each immediately followed by a 30-second rinse in DI water. After daily treatments 1 and 3, samples were immersed for 1 hour in a 37 °C incubator in pH 7.0 artificial saliva (2.20 g/L gastric mucin, 1.45 mmol/L calcium dichloride dihydrate, 5.42 mmol/L potassium dihydrogen phosphate, 6.50 mmol/L sodium chloride, 14.94 mmol/L potassium chloride)^37^ with the addition of 0.25 g/L amylase (alpha-amylase from *Bacillus subtilis*, MP Biomedicals) to provide amylase activity comparable to clinical levels.^38^ After daily treatment 2, samples were immersed for 4 hours in a pH 5.0 lactic acid demineralization gel (0.1 M aged lactic acid, 4.1 mM calcium dichloride dihydrate, 8 mM potassium dihydrogen phosphate, and 1% w/v carboxymethyl-cellulose [Sigma C5013, high viscosity] at 37 °C, to simulate an acid challenge.^39^ After completion of daily treatment 4 and rinsing, samples were immersed in artificial saliva with amylase overnight (~17 hours).

#### Microcomputed tomographic analysis of natural caries lesions

Crowns of human teeth were evaluated with microcomputed tomographic (microCT) analysis on a Scanco μCT 50 system (Scanco Medical). A 4.4 μm voxel size, 55 KVp, 145 μA intensity, 0.36° rotation step (180° angular range), and a 1,000 ms exposure per view were used for the scans, which were performed in water. The Scanco μCT software (HP, DECwindows Motif 1.6) provided 3-dimensional reconstruction. Caries lesions seen on microCT scans were divided into treated vs untreated sections, then subdivided as a function of lesion depth into a superficial section (0.0–0.1 mm), a moderate depth section (0.1–0.25 mm), and a deep section (0.25–0.45 mm). The analysis included histograms and summary statistics.

#### LC Rinse fluorescence

After completion of the treatment protocol, teeth were reevaluated with LC Rinse and new images were evaluated using AI-assisted image analysis. Relative changes in fluorescence comparing pretreatment and posttreatment were calculated.

#### White light visual assessment

The white spot and the esthetic appearance of noncavitated caries lesions were evaluated with a qualitative analysis of collected micrographs by 2 masked examiners (N.A.J., L.-C.P.) using a condensed (3-point) Likert scale (deteriorated, unchanged, or improved).^40^ In cases of disagreement, a consensus score was established. Categorical distributions by treatment type were compared.

#### Statistical analyses

When applicable, blinding was used throughout our study to prevent potential bias. Samples were numbered, labeled, and assigned randomly to treatment groups. Treatment application was unblinded as treatments had different physical appearances. All data collection, assessments, and analyses were performed on samples in numeric order, with evaluators blinded to their treatment type. Data were analyzed with Stata Version 12 (StataCorp LLC). Qualitative categorical comparisons were evaluated using Fisher exact test. Comparisons of microCT results were evaluated with a multivariable regression analysis predicting percent remineralization as a function of depth by treatment type. Comparisons of LC Rinse labeled fluorescence were evaluated by analysis of variance. *P* < .05 was considered significant.

## Results

### Mineral release studies

**Figure 2:**
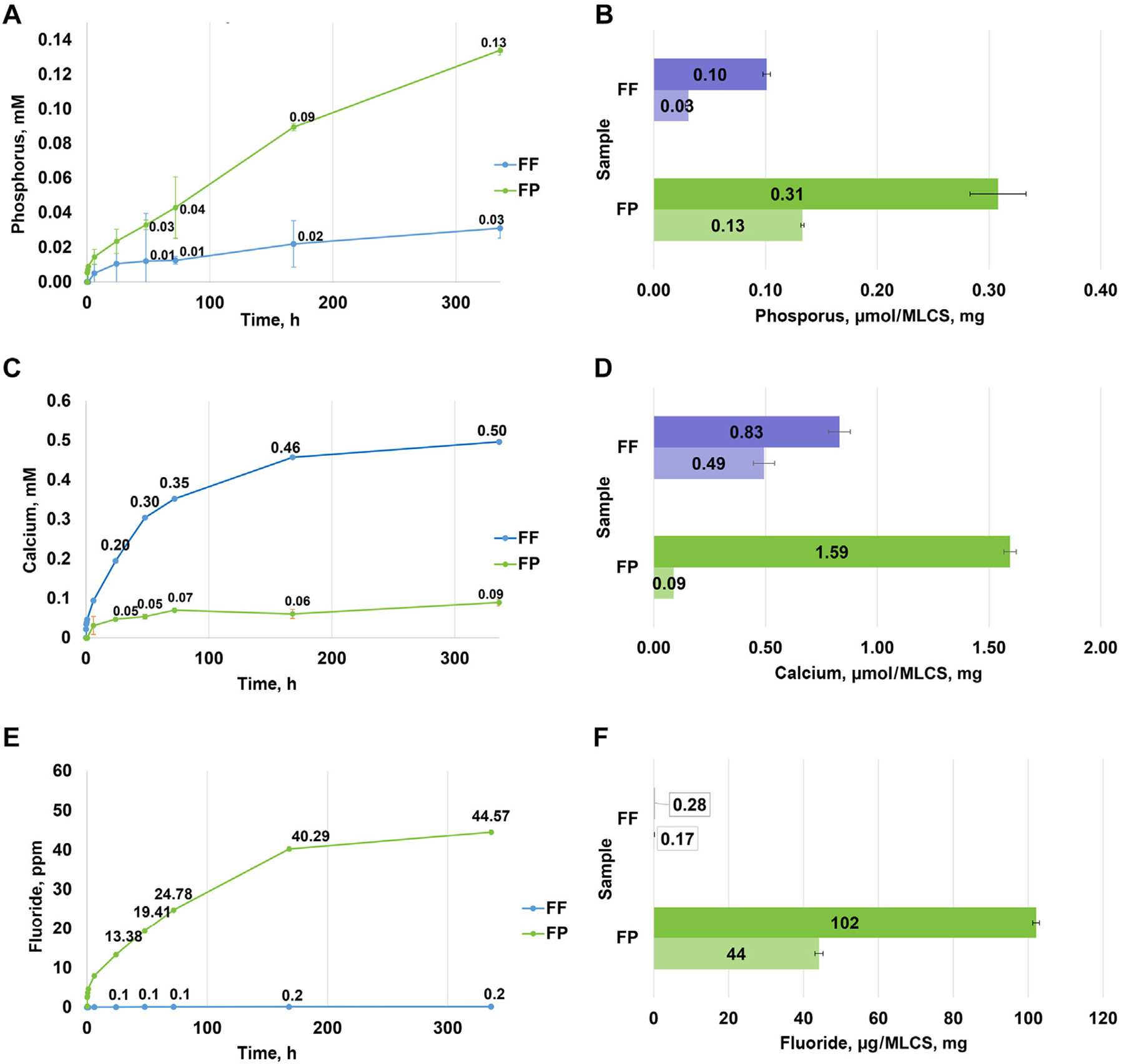
Release profiles from fluoride-free (FF) and fluoride-plus (FP) mineral-loaded cationic starch (MLCS) particles into deionized water at 37 °C and released mineral (lighter shading) compared with total mineral loading (darker shading) for phosphorus release (**A**) and loading (**B**), calcium release (**C**) and loading (**D**), and fluoride release (**E**) and loading (**F**), respectively.

Figure 2 shows mineral release profiles and loadings of the MLCS-FF and MLCS-FP particles. Release profiles of uncrystallized calcium, phosphate, and fluoride ions showed sustained release extending over weeks. Compared with the total mineral content of these particles, only a portion of the loaded minerals were released. Additional XRD characterization of these particles before release was performed (sFigure 1).

### Remineralization studies

#### MicroCT evaluation of mineral restoration

**Figure 3:**
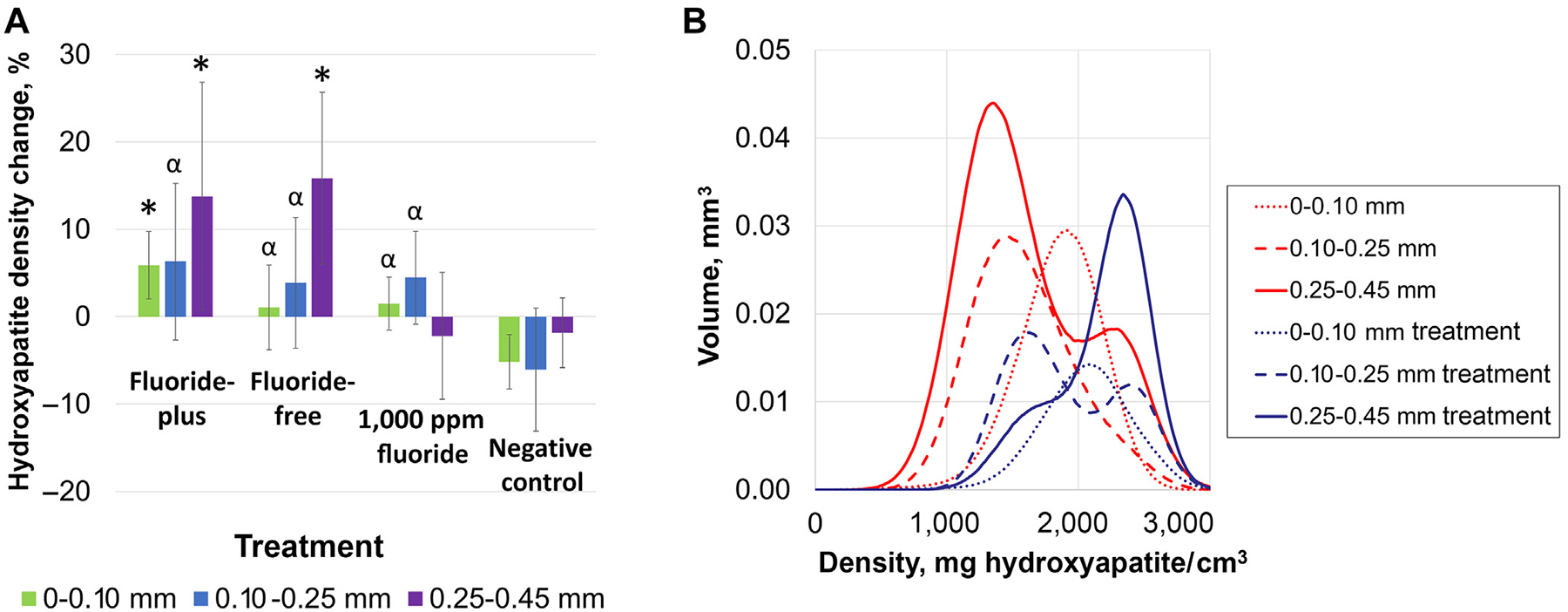
Analysis of natural lesions. **A**. Changes in mineral density after treatment as measured by microcomputed tomography. Statistically significant (*P* < .05) differences are denoted by the alpha symbol (α) for comparisons to negative control alone or an asterisk (*) for comparisons to both fluoride and negative controls. **B**. Example of microcomputed tomographic histograms for depth segments before (red) and after (blue) treatment with fluoride-plus mineral-loaded cationic starch particles.

MicroCT was used to summarize mineral density change by treatment arms as a function of depth from the tooth surface. Our analysis required using customized segmentation to adapt to the curved surfaces of the specimens, with depth segments of 0.00 through 0.10 mm, 0.10 through 0.25 mm, and 0.25 through 0.45 mm. A summary is shown in Figure 3A, in which the average differences in mineral density for each depth region were calculated by the treatment arm. The multivariable regression analysis found that all treatments (MLCS-FP, MLCS-FF, and 1,000 ppm fluoride control) were better than the negative control (DI water) for 0.0 through 0.1 mm (5.9% [3.9%], 1.1% [4.9%], 1.5% [3.0%], and −5.2% [3.1%] for FP, FF, fluoride, and DI water, respectively) and 0.1 through 0.25 mm depths (6.3% [9.0%], 3.8% [7.5%], 4.5% [5.3%], and 3.1% [7.0%] for FP, FF, fluoride, and DI water, respectively), and that the MLCS-FP particle composition was superior to the negative and fluoride controls for the surface portion 0.0 through 0.1 mm. The MLCS-FF and MLCS-FP particles yielded significantly superior mineral restoration for the subsurface zone (0.25–0.45 mm) than the 1,000 ppm fluoride control (13.8% [13.0%], 15.9% [9.4%], −2.2% [7.3%], and −1.8% [4.0%] relative hydroxyapatite density change for FP, FF, fluoride, and DI water, respectively). Calculated integrals of mineral deposition found relative lesion mineral restoration of 42.9% [41.4%], 38.6% [35.7%], 7.4% [22.9%], and −17.9% [22.7%] for FP, FF, fluoride, and DI water, respectively. A sample histogram for a caries lesion treated with the MLCS-FP particles, with the untreated region plotted for comparison, is shown in Figure 3B. This plot highlights that the superficial region of the lesion (dotted lines) saw limited differences in mineral density after treatment, whereas the lower mineral density subsurface areas (dashed and solid lines) saw a substantial increase in mineral density, confirming targeted delivery and subsurface mineralization.

#### LC fluorescence

**Figure 4:**
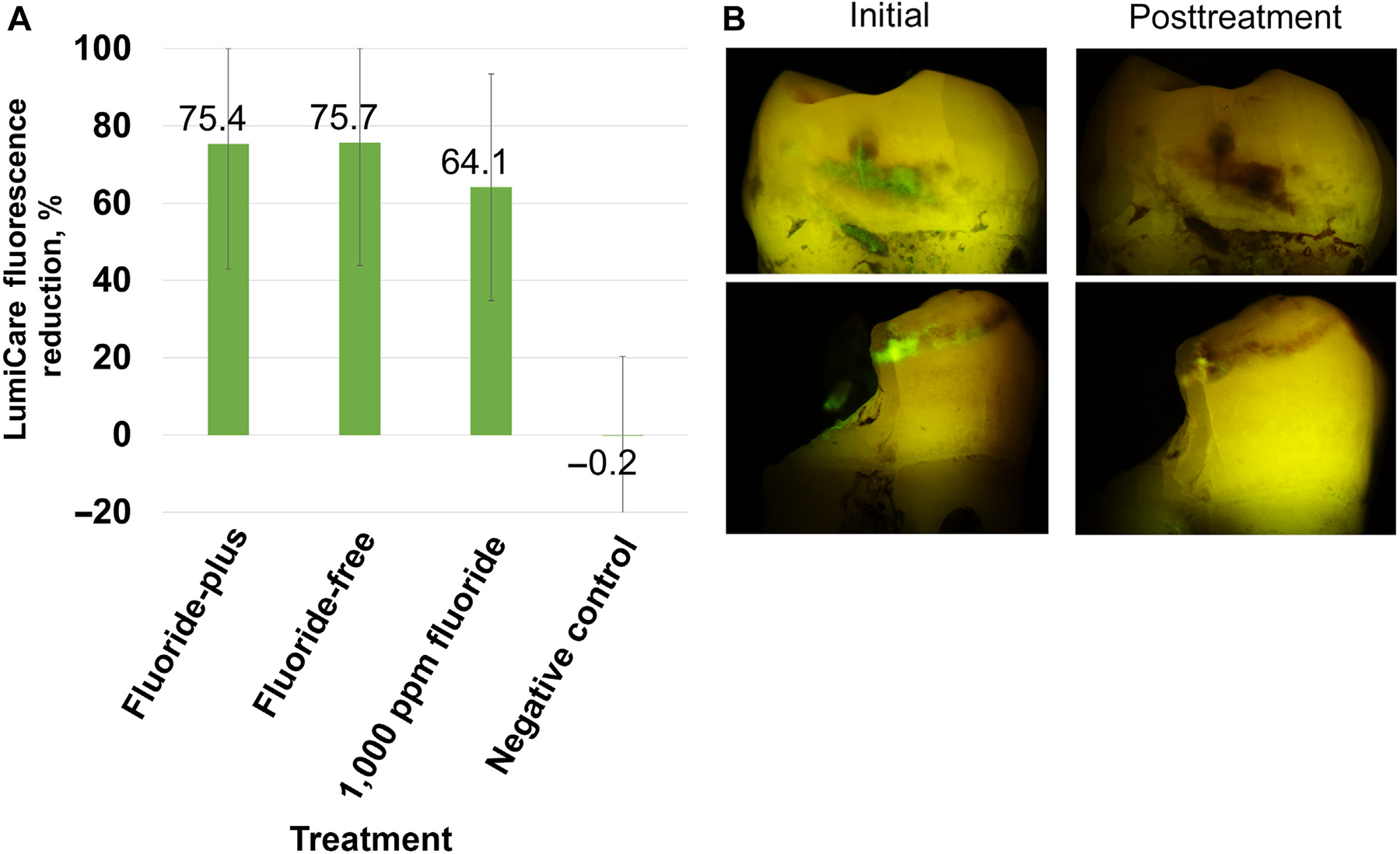
**A**. Quantitation of reduction in LumiCare Caries Detection Rinse (Greenmark Biomedical Inc) fluorescence pixels after treatment, as measured with machine-learning-based fluorescence analysis algorithm. The negative control was deionized water. **B**. Examples of LumiCare Caries Detection Rinse fluorescence before and after treatment, showing cases with complete and partial reduction in LumiCare fluorescence.

A summary of the change in fluorescence with LC Rinse and using the AI-based image analysis algorithm^36^ is shown in Figure 4A, with sample images in Figure 4B. LC Rinse fluorescence reduction was 75.4% [9.5%], 75.7% [8.6%], 64.1% [8.4%], and −0.2% [4.0%] for MLCS-FP, MLCS-FF, fluoride, and negative control, respectively. The analysis of variance found that MLCS-FP, MLCS-FF, and the fluoride control were all statistically superior to negative control (all *P* < .05).

#### White light visual assessment

**Figure 5:**
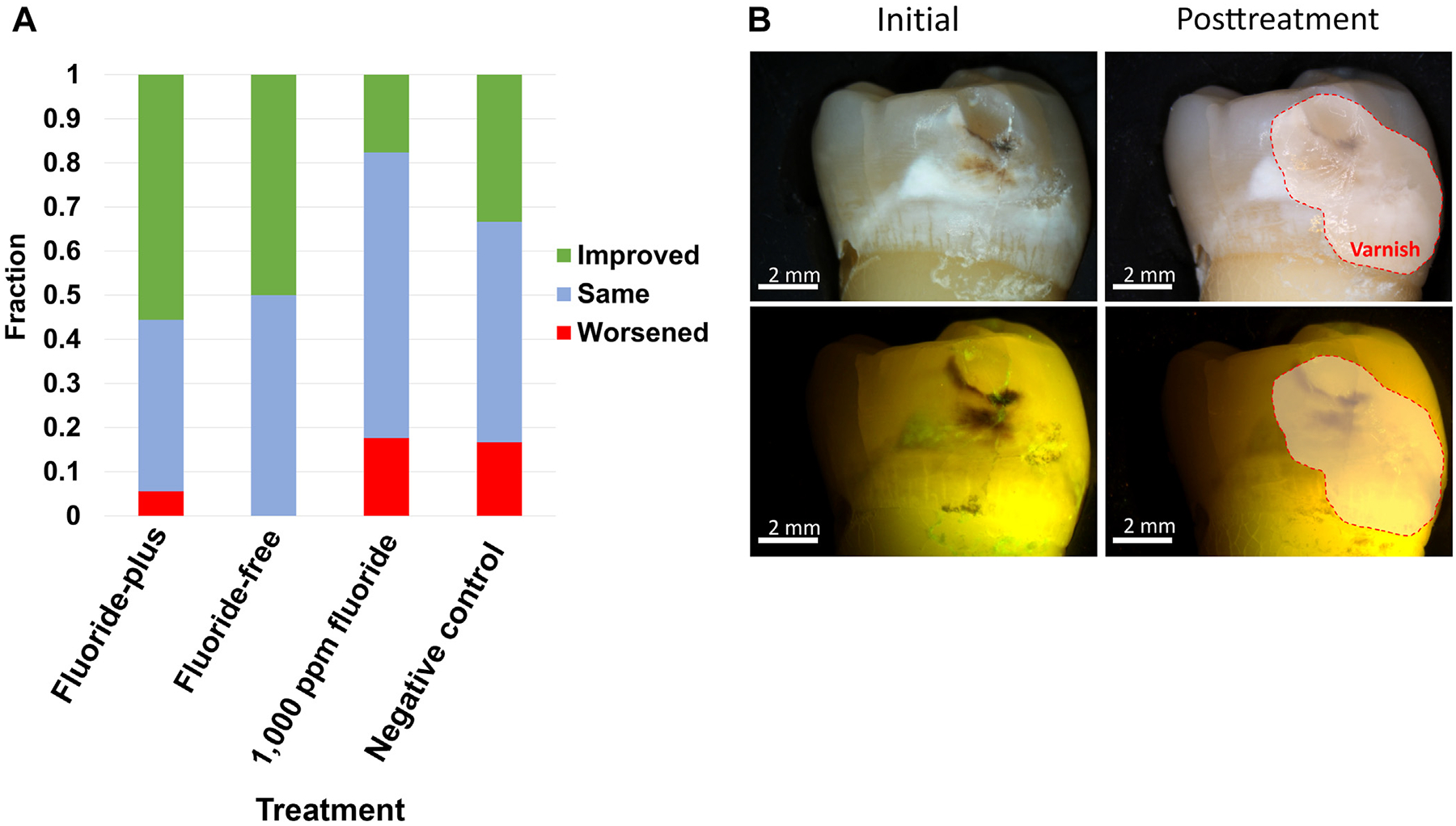
**A**. Blinded qualitative assessment of white-spot lesion esthetic change after treatment. The negative control was deionized water. **B**. Example of improved lesion appearance for tooth after treatment with fluoride-plus particles, with corresponding LumiCare Caries Detection Rinse (Greenmark Biomedicial Inc) fluorescence images. The varnish-coated half of the tooth region (untreated control) has been demarcated.

The distributions of qualitative categories after treatment are shown in Figure 5A, with an example of improved appearance after treatment with FP particles with both white light and corresponding LC Rinse fluorescence images shown in Figure 5B. Comparing MLCS-FF treated categorical outcomes to the 1,000 ppm fluoride and negative control, there was significance (*P* = .038 [Fisher exact test]).

## Discussion

We report a strategy for targeted subsurface treatment of early-stage noncavitated caries lesions in enamel using MLCS particles. We used natural lesions in extracted teeth and followed the recommendations of Coradin et al.^41^ in using multitechnique analysis of remineralization, with a preference toward microCT for the determination of mineral deposition quantification and spatiality.

The proposed subsurface targeting model (Figure 1B) requires 3 properties of guided mineral-loaded particles: (1) sufficiently small to permit diffusion or penetration into the active caries lesion subsurface; (2) having a driving force, or targeting moiety, for promoting accumulation within the subsurface; and (3) loading functional mineral ions that are released at destination. The first 2 properties, penetration and targeting, were validated in previous work, wherein cationic starch particles with sizes 150 through 350 nm were effective at targeting the subsurface of caries lesions and accumulating over the entirety of subsurface porosities.^29^

Our study extends findings to focus on the third property, which is releasing ions at the destination. Loading and release were both measured (Figure 2). The release profiles and XRD analysis (sFigure 1) show that the particles are loaded with uncrystallized (ie, functional) calcium, phosphate, and fluoride ions, which release slowly over weeks. Compared with total loading levels, only 5% through 60% of the minerals are released. Because the release of minerals is cotemporal, it is underestimated because of the precipitation of calcium, phosphate, and fluoride after release from the particle but before escape from the dialysis membrane. In the oral environment, salivary amylase will degrade the starch, thereby further triggering mineral release. These experiments identified the importance of keeping the particles dry until use to prevent premature mineral release and crystallization. The fluoride loading in the MLCS-FP particles, designed to be 1,000 ppm fluoride in a 1% w/w dispersion of the particles in saliva, was verified in this testing. This is the fluoride concentration allowable in over-the-counter toothpaste products in the United States as permitted by the US Food and Drug Administration’s anticaries fluoride monograph, and it was also the concentration used in the fluoride control for the subsequent remineralization study.

The hypothesized benefits of preferential mineralization of the lesion subsurface include treatment efficacy, esthetic benefits, and resilience. As an in vitro test system, natural smooth-surface noncavitated caries lesions were selected by an ICDAS-trained cariologist. These lesions were evaluated for lesion porosity or activity using LC Rinse, confirming positive or negative fluorescence by visualization and imaging. Remineralization was evaluated by microCT with depth-segmented histograms comparing the efficacy of treatment arms. sFigure 2 shows average mineral density depth profiles of untreated natural lesions, validating higher mineral density in the surface zone (0.00–0.10 mm) overlying a more porous subsurface lesion (0.10–0.25 mm and 0.25–0.45 mm). Supporting the targeted treatment model, both FP and FF compositions of MLCS particle treatments resulted in significantly greater subsurface remineralization than the 1,000 ppm sodium fluoride and negative (DI water) controls (Figure 3). Integration calculations of total mineral remineralization found that MLCS-FP and MLCS-FF treatments restored approximately 5.8 or 5.2 times more mineral than fluoride control, respectively. MLCS-FP particles showed improved surface zone remineralization relative to the 1,000 ppm fluoride control, which may be due to the sustained release and persistence of fluoride when released from the particles. All treatments (MLCS-FP, MLCS-FF, 1,000 ppm fluoride) were superior to the negative control.

After the 20-day remineralization protocol, LC Rinse fluorescence imaging and analysis were repeated. Both MLCS particle compositions and the fluoride control treatment significantly decreased lesion fluorescence (Figure 4). This could be because the lesions were sealed or arrested, from a surface perspective, or that simply all available negative surface charges were quenched after mineral precipitation throughout the entirety of the caries lesion. Furthermore, the white-spot esthetic of noncavitated caries lesions (Figure 5) benefited from targeted mineral treatment compared with 1,000 ppm fluoride and negative controls, though complete disappearance of the white-spot lesions was not observed. Treatment duration was potentially insufficient, though surface porosity was reduced to the point of rendering the lesion arrested (consistent with reduced fluorescence by LC Rinse).

Regarding published literature, it was difficult to compare our study using natural lesions with most in vitro caries remineralization studies that use artificial lesions. Artificial lesions are approximately an order of magnitude shallower than the lesions used in our study and may differ substantially in lesion mineral distribution and chemical composition; the use of natural lesions is more meaningful in showing subsurface remineralization that is benchmarked in our study to fluoride and negative controls.^41,42^

The results of our study are limited because of its in vitro nature. Additional testing is required to understand the clinical effect of this treatment strategy. The use of biological samples and natural lesions can result in high variability. Our study design and analysis attempted to mitigate heterogeneity through inclusion and exclusion criteria and then by comparing treatment responses with model controls and individual sample controls. Future research is required to evaluate clinical remineralization treatment efficacy. Treatment effects in vivo may show greater variability in clinical outcomes as expected with noncavitated caries lesions and diverse patient oral hygiene contexts. MicroCT analysis of natural caries lesions was simplified with set depth segments that covered most enamel-limited lesions, so treatment impact for lesions deeper than 0.45 mm is not directly known. In addition, only smooth-surface lesions were evaluated to simplify the analysis. LC Rinse fluorescent particles are effective at illuminating active caries lesions on all tooth surfaces.^31^ Therefore, a similar treatment effect is anticipated for MLCS particles on other tooth surfaces. Treatment resiliency was indirectly measured with all samples undergoing periodic acid cycling throughout the treatment process, and all treated samples showed benefit or a protective element compared with the DI water negative control. This may be further assessed with a nonconcurrent acid challenge.

Although our study was performed with guided particles containing calcium, phosphate, and fluoride, the principles of targeted delivery to caries lesions are applicable to other therapeutics, including antibiotic or antimicrobial agents,^43^ other mineral sources such as casein phosphopeptide–ACP^44^ or nanohydroxyapatite,^45^ or other remineralization aids such as peptide P11–4.^46^ There may be synergistic benefits of combining untargeted and targeted delivery approaches for caries treatment or for treatment of other oral diseases.

## Conclusions

Within the limitations of an in vitro study, potential benefits were shown when using guided mineral-loaded particles for subsurface remineralization of early-stage noncavitated caries lesions. MLCS showed the capability to deliver minerals preferentially to the negatively charged caries lesion subsurface. This is in stark contrast to untargeted therapies that predominantly remineralize and seal the upper surface layer without restoring the dominant subsurface caries lesion in enamel. A model for this targeted treatment strategy was presented, and advantages were proposed compared with traditional surface treatments, including treatment efficacy and lesion esthetics. Ongoing development of this treatment concept and future in vivo clinical testing can further validate this approach to noninvasive caries management.

## Supplementary Material

1

## Why Is This Important?

Noninvasive treatments for early-stage noncavitated caries act primarily at the lesion surface, which limits efficacy. In this study, the authors explore an alternative approach to remineralization treatment using targeted submicrometer particles to deliver calcium, phosphate, and fluoride preferentially to the subsurface porosities of noncavitated caries lesions. In vitro results indicated that targeted particles could improve treatment efficacy with increased subsurface mineralization, which leads to better esthetics, greater resilience to new caries processes, and improved long-term outcomes. Treatments based on these concepts may provide dentists and their patients with better tools to manage and prevent caries noninvasively.
