# Supplementary material for: Targeted enamel remineralization with mineral-loaded starch particles
Source: JADA Found Sci. Author manuscript; Available in PMC 2024 Dec 31. (PMC11687358; doi:10.1016/j.jfscie.2024.100041)
Supplement: Supplementary file 1 [file NIHMS2043437-supplement-1.pdf]

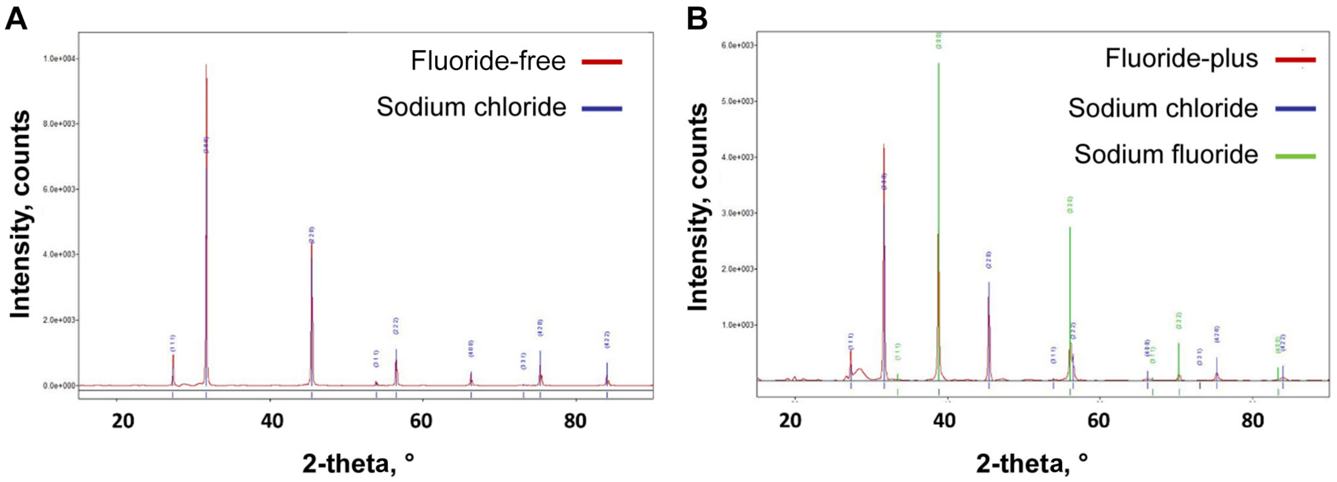

sFigure 1 X-ray diffraction spectra for fluoride-free mineral loaded starch particle powder (**A**) and fluoride-plus mineral-loaded starch nanoparticle powder (**B**). Recommended peak fitting is shown for each sample. Peak fitting using the x-ray diffraction (Rigaku Ultima) software identified only sodium chloride in the fluoride-free starch particle powder, and only sodium chloride and sodium fluoride in the fluoride-plus starch particles. Crystalline forms of calcium phosphate and calcium fluoride were not detected in this analysis.

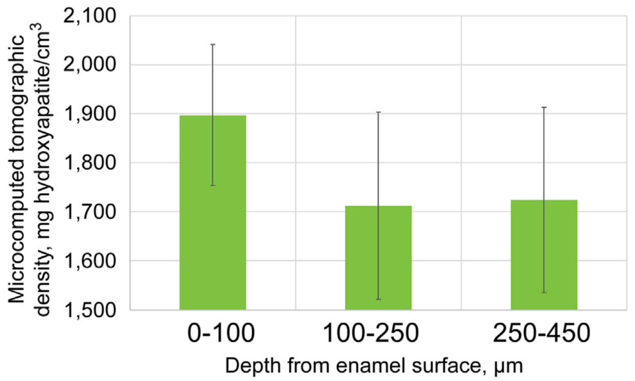

sFigure 2 Microcomputed tomographic mineral density for depth segments of untreated natural caries lesions, with higher mineral density in the superficial region than in the lesion subsurface. Caries lesions were half covered using a varnish coating so that the coated portion acts as a sample control and is representative of the untreated lesion. The average mineral density of untreated lesions shows that the mineral density structure of these lesions is consistent with the architectures described in Silverstone et al.[24] with a relatively intact surface bridge with higher mineral density overlying more substantial subsurface caries.
